# mTOR signaling in the arcuate nucleus of the hypothalamus mediates the anorectic action of estradiol

**DOI:** 10.1530/JOE-18-0190

**Published:** 2018-06-18

**Authors:** Ismael González-García, Pablo B Martínez de Morentin, Ánxela Estévez-Salguero, Cristina Contreras, Amparo Romero-Picó, Johan Fernø, Rubén Nogueiras, Carlos Diéguez, Manuel Tena-Sempere, Sulay Tovar, Miguel López

**Affiliations:** 1 Department of PhysiologyCiMUS, University of Santiago de Compostela-Instituto de Investigación Sanitaria, Santiago de Compostela, Spain; 2 CIBER Fisiopatología de la Obesidad y Nutrición (CIBERobn)Santiago de Compostela, Spain; 3 Hormone LaboratoryHaukeland University Hospital, Bergen, Norway; 4 KG Jebsen Center for Diabetes ResearchDepartment of Clinical Science, University of Bergen, Bergen, Norway; 5 Department of Cell BiologyPhysiology and Immunology, University of Córdoba, Córdoba, Spain; 6 Instituto Maimónides de Investigación Biomédica (IMIBIC)/Hospital Reina SofíaCórdoba, Spain; 7 FiDiPro ProgramUniversity of Turku, Turku, Finland

**Keywords:** estradiol, hypothalamus, food intake, mTOR, obesity

## Abstract

Current evidence suggests that estradiol (E2), the main ovarian steroid, modulates energy balance by regulating both feeding and energy expenditure at the central level, through the energy sensor AMP-activated protein kinase (AMPK). We hypothesized that the hypothalamic mechanistic target of rapamycin (mTOR) pathway, a well-established nutrient sensor and modulator of appetite and puberty, could also mediate the anorectic effect of E2. Our data showed that ovariectomy (OVX) elicited a marked downregulation of the mTOR signaling in the arcuate nucleus of the hypothalamus (ARC), an effect that was reversed by either E2 replacement or central estrogen receptor alpha (ERα) agonism. The significance of this molecular signaling was given by the genetic inactivation of S6 kinase B1 (S6K1, a key downstream mTOR effector) in the ARC, which prevented the E2-induced hypophagia and weight loss. Overall, these data indicate that E2 induces hypophagia through modulation of mTOR pathway in the ARC.

## Introduction

Besides the regulation of the reproductive function, estrogens have a key role in the central regulation of the energy homeostasis including both modulation of feeding behavior and energy expenditure (Mauvais-Jarvis* et al.* 2013, López & Tena-Sempere 2015, 2016, 2017, Palmer & Clegg 2015). Increased life expectancy implies that many women will live an increasing number of years in a state of ovarian insufficiency. This leads to a steady surge in obesity incidence reaching a staggering figure of greater than 70% in women older than 60 years (Flegal* et al.* 2010). Although the interrelationship between estrogen deficiency and obesity was the subject of some discussion, pooled data derived 107 trials showed that hormone-replacement therapy in menopausal patients led to reduced abdominal obesity, insulin resistance, new-onset diabetes, lipids, blood pressure, adhesion molecules and procoagulant factors in women without diabetes and reduced insulin resistance, as well as fasting glucose in women with diabetes (Salpeter* et al.* 2006), thus providing a cause–effect relationship between estrogen deficiency, obesity and metabolic complications.

From a mechanistic point of view, studies carried out in rodents showed that reduced levels of estradiol (E2) after ovariectomy (OVX) are associated with hyperphagia and decreased energy expenditure, leading to weight gain (Martínez de Morentin* et al.* 2014*a*, 2015). Moreover, variations in the magnitude of meals and body weight occur in rats throughout the estrous cycle, in parallel with changes in endogenous E2 levels (Blaustein & Wade 1976, Tritos* et al.* 2004, Mauvais-Jarvis* et al.* 2013, Martínez de Morentin* et al.* 2014*a*, López & Tena-Sempere 2015, 2017). The functional relevance of these data is supported by the fact that central administration of E2 elicits profound anorectic, catabolic and weight-reducing effects (Martínez de Morentin* et al.* 2014*a*, 2015).

At the central level, estrogen receptor alpha and beta (ERα and ERβ) are expressed in several hypothalamic nuclei with key roles in the regulation of energy balance, such as the arcuate (ARC), paraventricular (PVH) and ventromedial (VMH) (Simerly* et al.* 1990, Simonian & Herbison 1997, Voisin* et al.* 1997, Osterlund* et al.* 1998, Merchenthaler* et al.* 2004). Recent evidence has shown that E2 has a nucleus-specific action in the hypothalamus to modulate energy homeostasis, particularly within the ARC and the VMH. Thus, while most of the actions of estrogens on food intake take place through ERα in the ARC, its effect on energy expenditure is conducted through ERα in the VMH (Xu* et al.* 2011, Martínez de Morentin* et al.* 2014*a*, 2015). The molecular mechanisms mediating the effect of central estrogens are not totally understood, but recent data have demonstrated that E2 acting on ERα inhibits hypothalamic AMP-activated protein kinase (AMPK) and that genetic activation of this enzyme within the ARC reverses the anorectic action of E2 (Martínez de Morentin* et al.* 2014*a*).

The mechanistic target of rapamycin (mTOR) is an evolutionarily conserved serine-threonine kinase that acts as a cellular sensor of changes in growth factors, nutrients and oxygen (Wang & Proud 2009, Laplante & Sabatini 2012, Martinez de Morentin* et al.* 2014*b*). mTOR phosphorylates and modulates the activity of the serine/threonine ribosomal protein S6 kinase B1 (S6K1). In turn, S6K1 phosphorylates and activates S6, a ribosomal protein involved in protein translation (Wang & Proud 2009, Laplante & Sabatini 2012, Martinez de Morentin* et al.* 2014*b*). Specifically, hypothalamic mTOR signaling plays a key role in modulating energy balance by responding to nutrient availability and the hormonal milieu (Cota* et al.* 2006, 2008, Blouet* et al.* 2008, Mori* et al.* 2009, Martins* et al.* 2012, Varela* et al.* 2012). Furthermore, a link between mTOR and the gonadal axis has been reported. Specifically, hypothalamic mTOR controls puberty onset and gonadotropin secretion by regulation of Kiss1 (Roa* et al.* 2009, Roa & Tena-Sempere 2010). This evidence indicates that mTOR signaling could contribute to the functional coupling between energy balance and gonadal activation and function. However, despite this evidence, it remains unclear whether mTOR might mediate the central actions of estrogens on food intake. The aim of this study was to assess whether the anorectic actions of E2 are mediated by specific modulation of mTOR signaling in the ARC.

## Materials and methods

### Animals

Adult female Sprague–Dawley rats (250–300 g; Animalario General USC, Santiago de Compostela, Spain) were used for the experiments. The experiments were performed in agreement with ‘International Law on Animal Experimentation’ and were approved by the USC Ethical Committee (Project ID 15010/14/006). The animals were housed with an artificial 12-h light (08:00–20:00)/12-h darkness cycle, under controlled temperature and humidity conditions and allowed free access to standard laboratory chow and tap water. For all the procedures, except during the washout period after OVX, the animals were caged individually.

### Ovariectomy

Rats were bilaterally OVX or sham-operated, in which each ovary was exposed but not tied or dissected, as previously described (Martínez de Morentin* et al.* 2014*a*, 2015, Skrede* et al.* 2017). All treatments (central or peripheral) on OVX rats were carried out 2 weeks after surgery to ensure a total washout of ovarian hormones, as previously reported (Martínez de Morentin* et al.* 2014*a*, 2015, Skrede* et al.* 2017).

### Determination of estrous cycle

Female rats were monitored for estrous cycle by daily vaginal cytology, and only rats with at least two consecutive regular 4-day estrous cycles were used in expression analyses, as previously reported (Martínez de Morentin* et al.* 2014*a*).

### Peripheral and central treatments

For the experiments with E2 replacement, OVX rats received a daily subcutaneous (SC) injection of estradiol benzoate (2 µg dissolved in 100 µL of sesame oil; both from Sigma) or vehicle (100 µL of sesame oil; control rats) during 5–11 days (Vigo* et al.* 2007, Roa* et al.* 2009, Martínez de Morentin* et al.* 2014*a*, 2015, Skrede* et al.* 2017).

For the central treatments, intracerebroventricular (ICV) cannulae were stereotaxically implanted under ketamine/xylazine anesthesia, as previously described (Varela* et al.* 2012, Martínez de Morentin* et al.* 2014*a*, 2015, Martínez-Sánchez* et al.* 2017). Animals were individually housed and used for experimentation 4 days later. For the central estrogen receptor agonists setting, OVX rats received one daily injection of the selective ERα agonist 4,4′,4″-(4-propyl-[1H]-pyrazole-1,3,5-triyl)trisphenol (PPT, 5 nmol dissolved in 5 µL of DMSO; TOCRIS Bioscience, Bristol, UK) or vehicle (5 µL of DMSO; control rats) for 5 days (Sanchez-Criado* et al.* 2004, 2006, Roa* et al.* 2008, Martínez de Morentin* et al.* 2014*a*, 2015). For the central leucine (Leu) experiments, OVX rats received one daily injection of Leu (10 nmol dissolved in 5 µL of saline) (Laeger* et al.* 2014) or vehicle (5 µL of saline) for 6 days.

### Stereotaxic microinjection

Rats treated with vehicle or E2 were placed in a stereotaxic frame (David Kopf Instruments, Tujunga, CA, USA) under ketamine/xylazine anesthesia. The ARC was targeted using a 25-gauge needle (Hamilton, Reno, NV, USA). The ARC injections were bilaterally directed to the following stereotaxic coordinates: −2.8 mm posterior (one injection was performed in each ARC), ±0.3 mm lateral to bregma and 10.2 mm dorso-ventral, as previously reported (Varela* et al.* 2012, Contreras* et al.* 2014, Martínez de Morentin* et al.* 2014*a*, Martínez-Sánchez* et al.* 2017). Adenoviral vectors (SignaGen, Rockville, MD, USA) encoding green fluorescence protein (GFP, used as control), S6K1 dominant negative (S6K1-DN; at 10^10^ pfu/μL) or S6K1 constitutively active (S6K1-CA; at 5 × 10^10^ pfu/μL) isoforms, were delivered at a rate of 200 nL/min for 5 min (1 μL/injection site) as previously reported (Varela* et al.* 2012, Contreras* et al.* 2014, Martínez de Morentin* et al.* 2014*a*, Martínez-Sánchez* et al.* 2017). The adenoviral and E2 treatments started at the same time. Direct detection of GFP fluorescence was performed after perfusion of the rats and detected with a fluorescence microscope Olympus IX51 at 4× augmentation.

### Sample processing

Rats were killed by cervical dislocation and decapitation. From each animal, the ARC was collected and immediately homogenized on ice to preserve phosphorylated protein levels. Those samples and the serum were stored at −80°C until further processing. Dissection of the ARC was performed by micro-punch procedure under the microscope, as previously described (Varela* et al.* 2012, Contreras* et al.* 2014, Martínez de Morentin* et al.* 2014*a*, Martínez-Sánchez* et al.* 2017). The specificity of the ARC dissections was confirmed by analyzing the protein levels of the specific marker proopiomelanocortin (POMC; data not shown).

### Hormone measurements

Circulating E2 levels were determined using a commercial ultra-sensitive RIA kit (Beckman Coulter, Brea, CA, USA). The sensitivity of the assay was 2.2 pg/mL, and the intra- and inter-assay CVs were 8.9% and 12.2%, respectively.

### Western blotting

ARC protein lysates were subjected to SDS-PAGE, electro-transferred on a PVDF membrane and probed with the following antibodies: mTOR, pmTOR Ser2448, S6K1, pS6K1 Thr389, S6, pS6 Ser235/236 (Cell Signalling, Danvers, MA, USA), and β-actin (Abcam, Cambridge, UK) as previously described (Varela* et al.* 2012, Martínez de Morentin* et al.* 2014*a*, 2015, Martínez-Sánchez* et al.* 2017). Values were expressed relative to β-actin protein levels. Autoradiographic films were scanned and the bands signal was quantified by densitometry using ImageJ-1.33 software (NIH, Bethesda, MD, USA). Representative images for all proteins are shown and each protein was corrected by its own internal β-actin control. In the gel images, all the bands for each picture come always from the same gel, but they may be spliced for clarity, as indicated in the figure legends.

### Statistical analysis

Data are expressed as mean ± s.e.m. Protein data are expressed relative (%) to control (Sham, OVX, vehicle or GFP treated) rats. Statistical significance is determined by Student *t* test when two groups is compared or ANOVA and *post hoc* Bonferroni test when more than two groups are compared. *P* < 0.05 is considered significant. The number of animals used in each experimental setting and analysis are specified in each figure legend.

## Results

### Lack of ovarian function decreases mTOR signaling in the ARC

**Figure 1:**
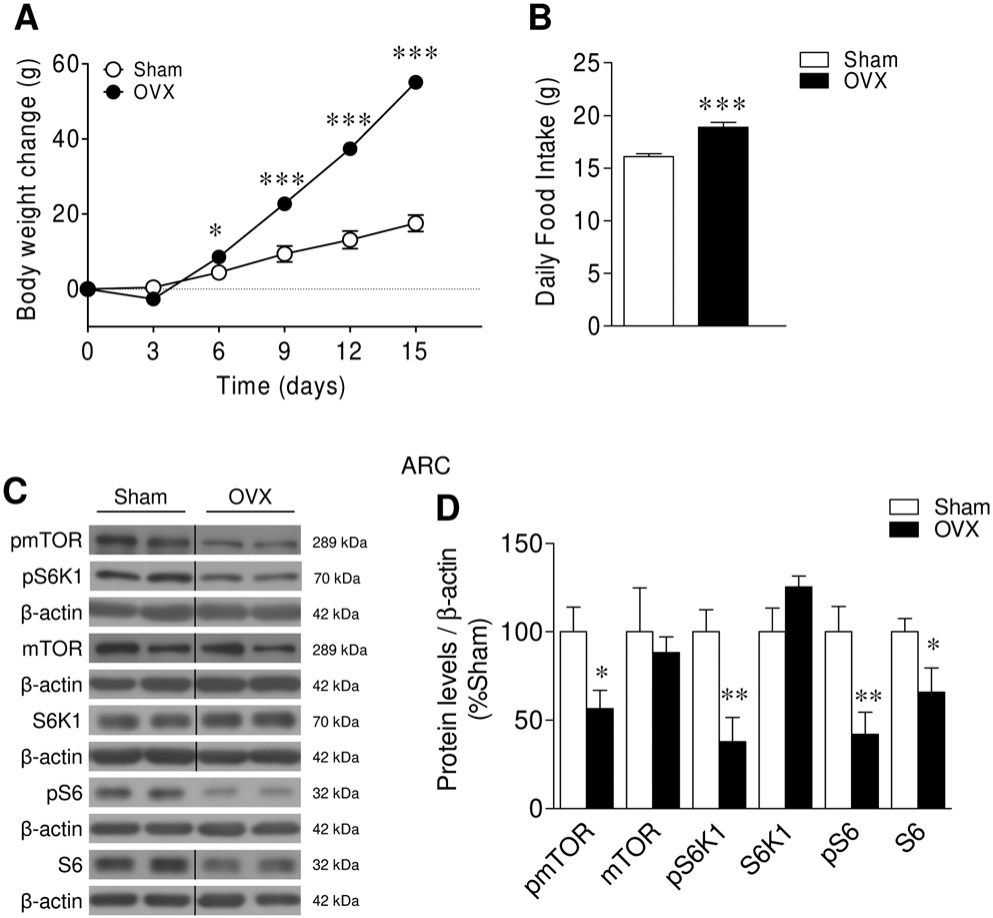
Effect of ovariectomy on energy balance and mTOR pathway within the ARC. (A) Body weight change, (B) daily food intake, (C) representative Western blot auto-radiographic images and (D) ARC protein levels of mTOR pathway of Sham rats or OVX rats. *n* = 30–32 animals per group for body weight and food intake data; *n* = 7 animals per group for Western blot data. All data are expressed as mean ± s.e.m. *, ** and ***P < 0.05, 0.01 and 0.001 vs Sham. For the Western blot analysis, representative images for all proteins are shown. In the gel images, all the bands for each picture come always from the same gel, but they may be spliced for clarity; in such case, this is depicted as vertical black lines.

OVX rats gained significantly more weight and developed marked hyperphagia (Fig. 1A and B). OVX rats showed the expected decrease in circulating E2 levels (Sham: 21.75 ± 2.68 pg/mL vs OVX: 9.35 ± 0.49 pg/mL; *P* < 0.001; *n* = 9 rats/group) confirming the efficiency of the OVX procedure. Our data showed that OVX induced a marked inactivation of mTOR signaling, as demonstrated by decreased protein levels in the ARC of phosphorylated (active) mTOR (pmTOR) at Ser2448, and its downstream targets, namely, pS6K1 at Thr389 and pS6 at Ser235/236 (Fig. 1C and D). These data suggested that ovarian function regulated mTOR signaling in the ARC.

### Estradiol increases mTOR signaling in the ARC

**Figure 2:**
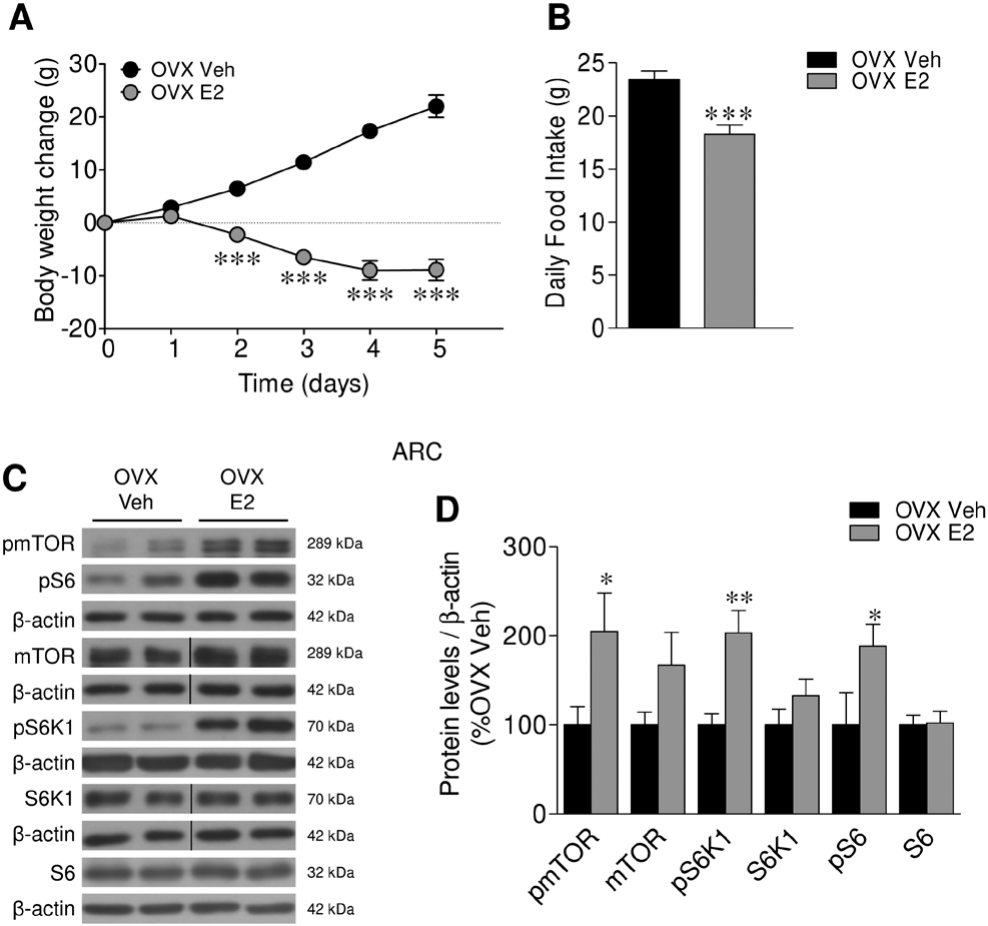
Effect of E2 replacement on energy balance and mTOR pathway within the ARC in OVX rats. (A) Body weight change, (B) daily food intake, (C) representative Western blot auto-radiographic images and (D) ARC protein levels of mTOR pathway of OVX rats SC treated with vehicle or E2. *n* = 8–10 animals per group for body weight and food intake data; *n* = 6–10 animals per group for Western blot data. All data are expressed as mean ± s.e.m. *, ** and ***P < 0.05, 0.01 and 0.001 vs OVX vehicle. For the Western blot analysis, representative images for all proteins are shown. In the gel images, all the bands for each picture come always from the same gel, but they may be spliced for clarity; in such case, this is depicted as vertical black lines.

To gain more insight in the effect of ovarian function on mTOR, we analyzed the effects of E2 replacement on mTOR signaling in the ARC of OVX rats. Our data showed that E2 administration to OVX rats induced weight loss and reduced feeding (Fig. 2A and B), associated with activation of the ARC mTOR pathway, demonstrated by elevated levels of pmTOR, pS6K1 and pS6 in that nucleus (Fig. 2C and D). To further explore the physiological relevance of our findings, we investigated the modulation of mTOR pathway of rats in proestrous, a stage of the cycle with elevated E2 levels (Martínez de Morentin* et al.* 2014*a*, 2015, Skrede* et al.* 2017). Our data showed that when compared with OVX animals, rats at proestrous exhibited a higher activation of mTOR signaling in the ARC (Supplementary Fig. 1A, see section on supplementary data given at the end of this article). This evidence suggested that endogenous E2 levels were likely physiological regulators on hypothalamic mTOR.

### ERα agonism increases mTOR signaling in the ARC

**Figure 3:**
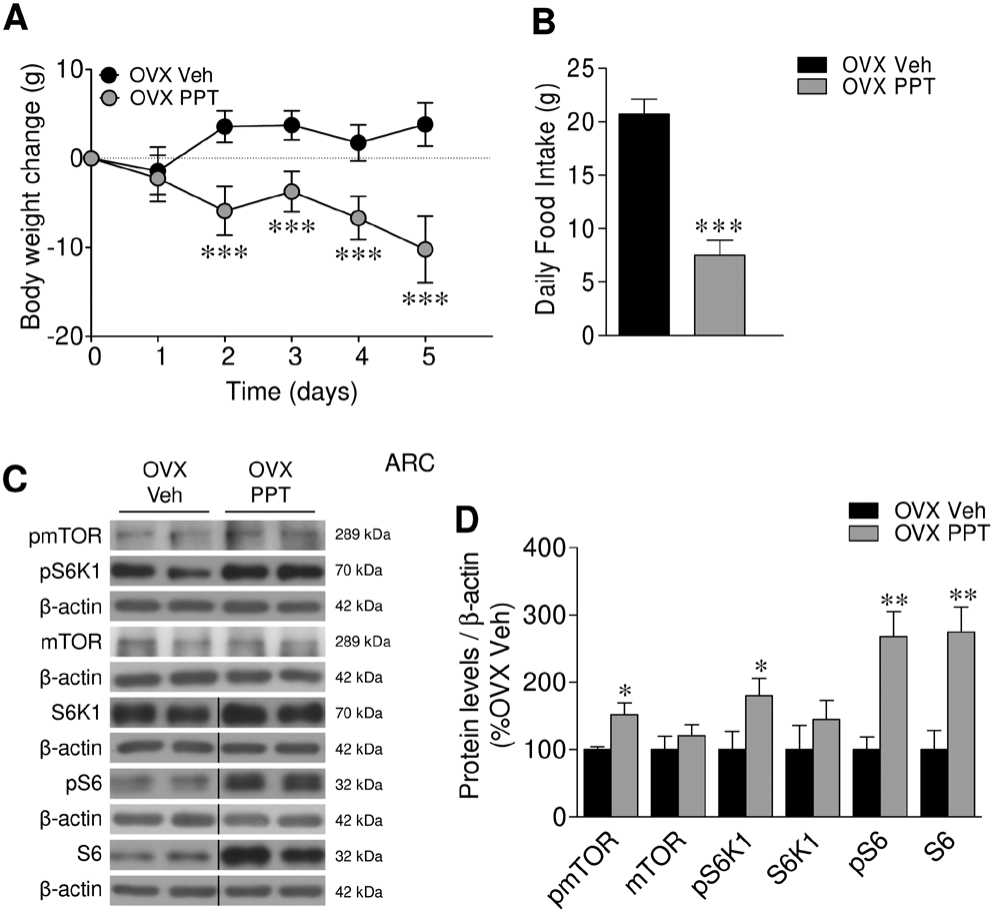
Effect of central PPT on energy balance and mTOR pathway within the ARC in OVX rats. (A) Body weight change, (B) daily food intake, (C) representative Western blot auto-radiographic images and (D) ARC protein levels of mTOR pathway of OVX rats ICV treated with vehicle or PPT. *n* = 7–12 animals per group for body weight and food intake data; *n* = 7 animals per group for Western blot data. All data are expressed as mean ± s.e.m. *, ** and ***P < 0.05, 0.01 and 0.001 vs OVX vehicle. For the Western blot analysis, representative images for all proteins are shown. In the gel images, all the bands for each picture come always from the same gel, but they may be spliced for clarity; in such case, this is depicted as vertical black lines.

Compelling evidence has demonstrated that the anorectic effect of E2 is mediated by ERα in the ARC (Xu* et al.* 2011); therefore, we aimed to investigate whether the effect of E2 on mTOR signaling was mediated by this receptor. ICV administration of the specific ERα agonist, PPT (Sanchez-Criado* et al.* 2004, 2006, Roa* et al.* 2008, Martínez de Morentin* et al.* 2014*a*, 2015), to OVX rats mostly recapitulated the effects of E2 by eliciting weight loss and anorexia (Fig. 3A and B), with concomitant activation of the mTOR pathway in the ARC (Fig. 3C and D), being the slight differences observed likely related to the route of administration (SC E2 vs ICV PPT). Overall, these data demonstrated that the anorectic effect of E2 in the ARC was associated to increased mTOR signaling in this hypothalamic nucleus, likely via the ERα receptor.

### Central activation of mTOR with leucine recapitulates the effect of E2

**Figure 4:**
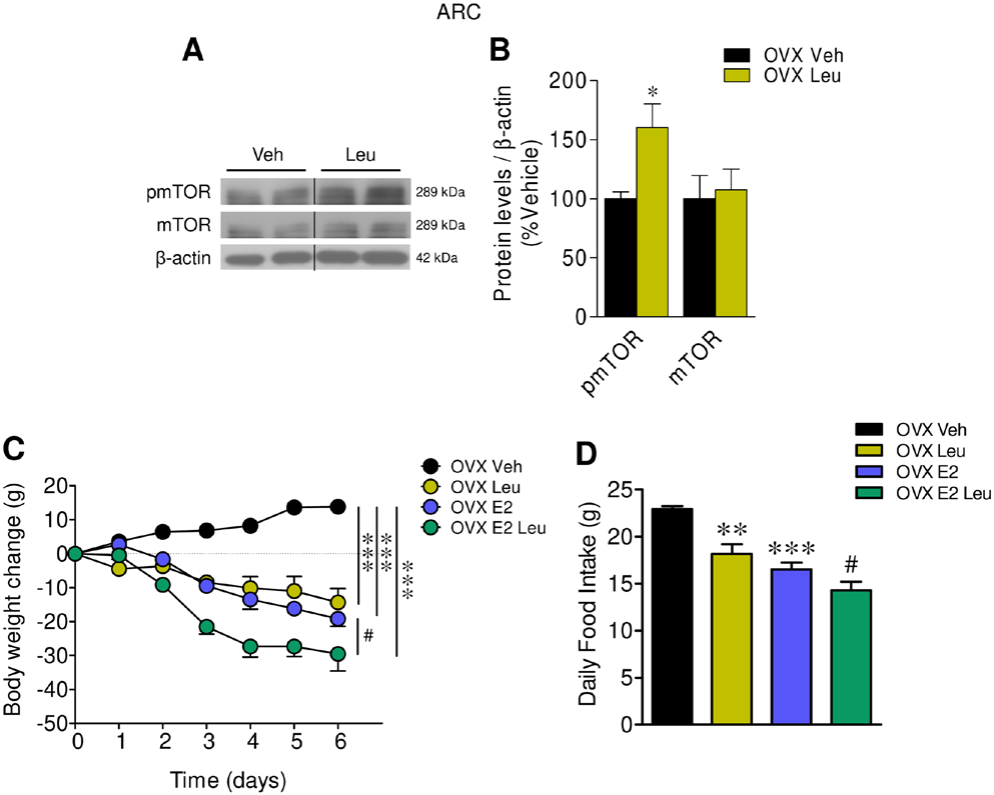
Effect of central Leu and E2 on energy balance in OVX rats. (A) Representative Western blot autoradiographic images and (B) densitometry measures of protein levels of pmTOR and mTOR in the ARC of OVX rats ICV treated with vehicle or Leu. (C) Body weight change and (D) daily food intake of OVX rats ICV treated with vehicle or Leu and/or SC treated with vehicle or E2; *n* = 6 animals per group for body weight, food intake and Western blot data. All data are expressed as mean ± s.e.m. *, ** and ***P < 0.05, *P* < 0.01 and 0.001 vs OVX vehicle; ^#^ *P* < 0.05 vs OVX E2. For the Western blot analysis, representative images for all proteins are shown. In the gel images, all the bands for each picture come always from the same gel, but they may be spliced for clarity; in such case, this is depicted as vertical black lines.

Given that E2- and PPT-induced anorexia in association to increased mTOR signaling in the ARC of OVX rats, we next aimed to investigate whether activation of this pathway impacted feeding in OVX rats. Our data showed that central administration of Leu, a well-established activator of mTOR (Cota* et al.* 2006, Laeger* et al.* 2014) increased the levels of pmTOR in the ARC (Fig. 4A and B), and decreased body weight (*P* < 0.001; *F* = 23.67) and food intake (*P* < 0.001; *F* = 18.88) in OVX rats in a similar magnitude to E2 (Fig. 4C and D).

### Inhibition of S6K1 in the ARC reversed the anorectic effect of E2

**Figure 5:**
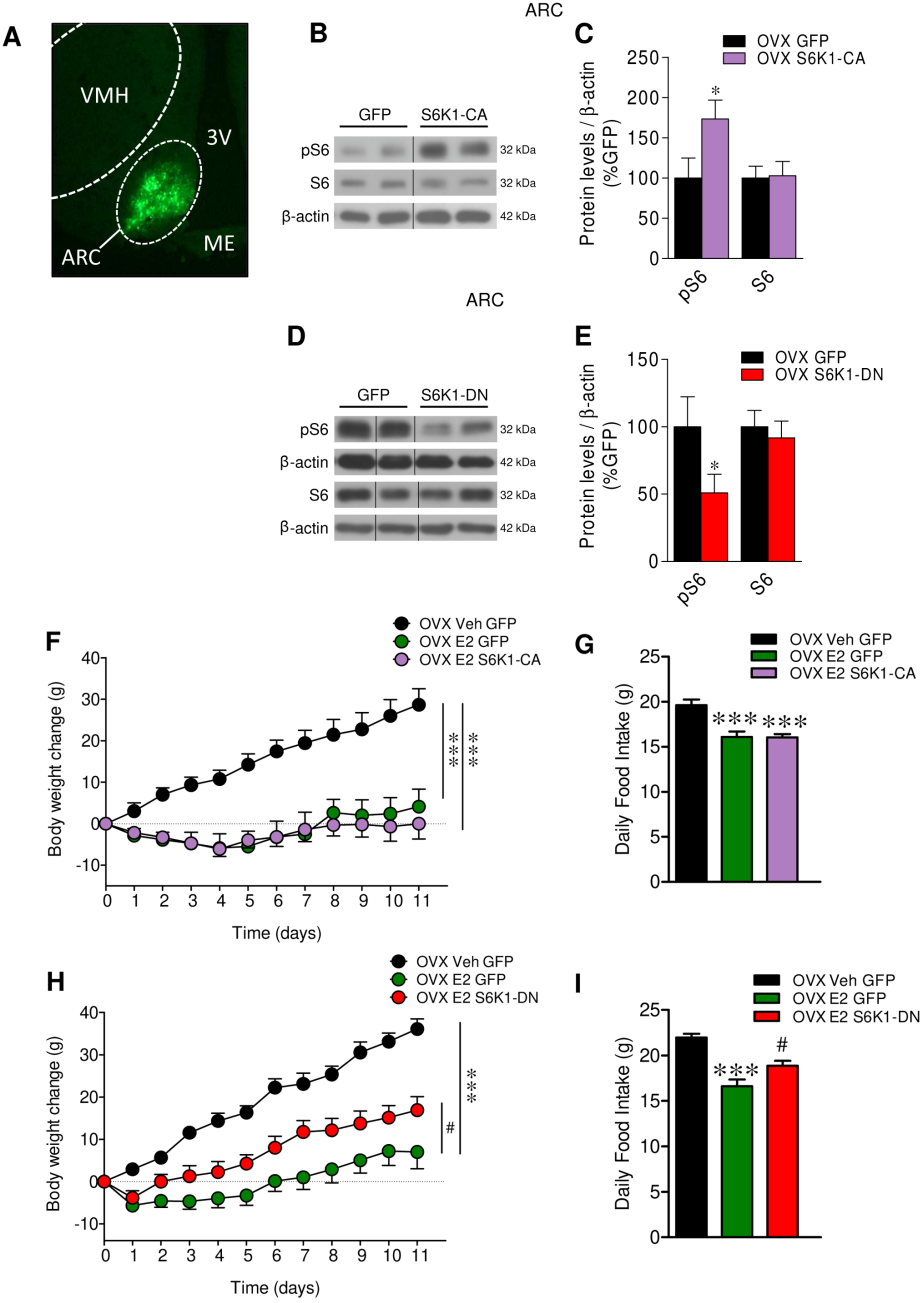
Effect of activation and down-regulation of mTOR pathway on the central actions of E2 on energy balance in OVX rats. (A) Direct fluorescence of GFP, (B and D) representative Western blot auto-radiographic images and (C and E) protein levels of pS6 and S6 in the ARC of OVX rats stereotaxically treated in the ARC with adenoviruses encoding GFP, S6K1-CA or S6K1-DN. (F) Body weight change and (G) daily food intake of OVX rats stereotaxically treated in the ARC with adenoviruses encoding GFP or S6K1-CA and SC treated with vehicle or E2. (H) Body weight change and (I) daily food intake of OVX rats stereotaxically treated in the ARC with adenoviruses encoding GFP or S6K1-DN and SC treated with vehicle or E2. *n* = 8–11 animals per group for body weight and food intake data; *n* = 7 animals per group for Western blot data. All data are expressed as mean ± s.e.m. * and ***P < 0.05 and 0.001 vs GFP or OVX Veh GFP; ^#^ *P* < 0.05 vs OVX E2 GFP. For the Western blot analysis, representative images for all proteins are shown. In the gel images, all the bands for each picture come always from the same gel, but they may be spliced for clarity; in such case, this is depicted as vertical black lines.

To further investigate the role of mTOR signaling on the anorectic effect of E2, we targeted S6K1, a direct downstream target of mTOR by using adenoviruses encoding either S6K1-CA or S6K1-DN or control adenovirus expressing GFP in the ARC. Injection in the ARC was assessed by visualization of GFP expression (Fig. 5A) and the efficiency of the treatment by assessing the protein levels of pS6, the downstream target of S6K1. S6K1-CA treatment increased, while S6K1-DN decreased, pS6 levels in the ARC, when compared with GFP controls (Fig. 5B, C, D and E). While administration of S6K1-CA in the ARC of E2-treated OVX rats decreased body weight (*P* < 0.001; *F* = 15.95) and reduced feeding (*P* < 0.01; *F* = 6.50) in a similar magnitude to E2 (Fig. 5F and G), S6K1-DN ameliorated the effect of simultaneously given E2 by increased food intake (*P* < 0.001; *F* = 15.98) and therefore body mass (*P* < 0.001; *F* = 20.33) (Fig. 5H and I). No changes in body weight were detected when vehicle-treated OVX rats were administered with S6K1-CA or S6K1-DN adenoviruses (Supplementary Fig. 2A and B). Overall, these data indicate that the anorectic effect of E2 is, in part, mediated by stimulation of mTOR signaling in the ARC.

## Discussion

Current evidence has demonstrated that E2’s anorectic effect is mediated by ERα signaling in the ARC (Xu* et al.* 2011, Martínez de Morentin* et al.* 2014*a*, 2015). However, the molecular mechanism explaining this effect remains unclear. Here, we show for the first time that mTOR signaling in the ARC is modulated by E2, and specifically by ERα agonism, an effect that mediates the anorectic actions of this estrogen.

Over the last decade, it has become clear that besides the classical neuropeptide networks, key molecular pathways regulate energy balance in the hypothalamus. Among them, energy sensors, such as AMPK (Kahn* et al.* 2005, López* et al.* 2016, López 2017) and nutrient sensors, such as mTOR (Cota* et al.* 2006, Blouet* et al.* 2008, Varela* et al.* 2012, Martinez de Morentin* et al.* 2014*b*) play a major role. We have recently documented the role of AMPK in the central actions of E2. Our data suggest that AMPK mediates the effects of E2 on energy balance in a dual manner; food intake is regulated via effects on AMPK in the ARC, whereas modulation of AMPK action in the VMH controls energy expenditure through sympathetic regulation of the brown fat (Martínez de Morentin* et al.* 2014*a*, 2015).

Whether mTOR signaling plays a role in the anorectic actions of central E2 is currently unknown, but the idea is supported by data showing that AMPK and mTOR pathways interact in the hypothalamus to modulate energy balance (Dagon* et al.* 2012, Varela* et al.* 2012) and also by the fact that the mTOR route mediates the effects on feeding of other peripheral signals such as thyroid hormones and ghrelin (Martins* et al.* 2012, Varela* et al.* 2012). Moreover, it is known that mTOR acts in the hypothalamus regulating the hypothalamus–pituitary–gonadal axis (HPG) (Roa* et al.* 2009). All this evidence led us to investigate the possible connection of the hypothalamic mTOR pathway with E2’s anorectic action.

Our data show that mTOR signaling is modulated by E2 acting at the central level, as demonstrated by the fact that E2 administration reversed the OVX-induced decrease in mTOR signaling in the ARC. One possible constraint of our study is the use of OVX rats, which are probably the most ‘*classical*’ model for the study of ovarian steroid actions (Martínez de Morentin* et al.* 2014*a*, 2015, Skrede* et al.* 2017). In this context, the major strength of this model, namely the lack of ovarian estrogens, it is also a limitation, which makes difficult to extrapolate the conclusions to a physiological context (the ovarian-intact, cycling female), perhaps apart from aging-related decline in ovarian function. To overrule this limitation, we analyzed the mTOR pathway in rats at the proestrous stage of the cycle, when the E2 levels are maximal (Martínez de Morentin* et al.* 2014*a*, 2015). Our data showed that mTOR signaling in the ARC is activated in proestrous when compared with OVX rats, suggesting that the mTOR pathway in the ARC is physiologically regulated by ovarian steroid milieu. Of course, other regulators of hypothalamic mTOR cannot be ruled out. For example, it is known that the increased adiposity that follows OVX is associated with increased leptinemia and insulinemia (Mauvais-Jarvis* et al.* 2013). Considering that both leptin and insulin are major modulators of hypothalamic mTOR (Dagon* et al.* 2012, Martinez de Morentin* et al.* 2014*b*), thus, the possible development of central leptin and/or insulin resistance could be a contributing factor.

One interesting fact is that the effect of E2 on mTOR signaling is mediated by ERα in this nucleus, since administration of the specific ERα agonist, PPT, mostly recapitulates (with slight differences in feeding, possible due to the protocol of administration: SC E2 vs ICV PPT) this action. This is of importance, because ERα is the primary ER isoform to modulate the anorectic properties of E2 at the hypothalamic level (Xu* et al.* 2011, Martinez de Morentin* et al.* 2015). Therefore, we hypothesized that hypophagia and subsequent weight loss after E2 or PPT administration might be mediated by specific modulation of mTOR signaling in the ARC. Our results show that either pharmacological activation of mTOR signaling following central administration of Leu or genetic activation of the downstream protein S6K1 with specifically delivery of S6K1-CA adenoviruses in the ARC prevented the OVX-induced hyperphagia and body weight gain. Importantly, the specific inhibition of the S6K1 in the ARC with S6K1-DN isoforms, partially blunted the anorectic effect of E2. These data demonstrate that the central actions of E2 on energy balance are at least partially mediated by the selective modulation of mTOR pathway through ERα and that this effect is placed in the ARC.

The molecular relevance of this evidence is intriguing. AMPK and mTOR function as major regulators of cellular metabolism that respond to changes in energy and nutrient status (Martinez de Morentin* et al.* 2014*b*, López* et al.* 2016). Both *in vitro* and *in vivo* results have demonstrated that activation of AMPK suppresses mTOR signaling (Bolster* et al.* 2002, Krause* et al.* 2002, Kimura* et al.* 2003, Saha* et al.* 2010) and also that S6K1 phosphorylates AMPK to mediate leptin’s action on feeding (Dagon* et al.* 2012). Furthermore, AMPK phosphorylates mTOR at Thr2446, inhibiting its function, which in turn decreases S6K1 phosphorylation (Cheng* et al.* 2004). Therefore, considering that AMPK in the ARC mediates the appetite-suppressive effect of E2 (Martínez de Morentin* et al.* 2014*a*), it would be tempting to speculate that both routes might act in this hypothalamic nucleus to coordinately regulate feeding and energy balance, a hypothesis that warrants further studies. In this sense, it will be critical to identify the ARC neuronal populations mediating that action. The most obvious candidate would be POMC neurons, which have been demonstrated to be critical for mediating E2’s anorectic effects (Xu* et al.* 2011, Martinez de Morentin* et al.* 2015). However, the well-described actions of E2 on specific glial cell populations, such as astrocytes (Azcoitia* et al.* 2011, Acaz-Fonseca* et al.* 2014, 2016), with a known role on energy balance (Garcia-Caceres* et al.* 2016), and the fact that the used S6K1 adenoviruses can also infect those cells, make astrocytes also interesting candidates to explore in further studies. The molecular underpinnings of that effect are also of interest for investigation. In this sense, the existence of membrane-initiated estrogen signaling effects in POMC neurons, involving for example mitogen-activated protein kinase (MAPK), phosphoinositide 3-kinase (PI3K) and protein kinase C (PKC) (López & Tena-Sempere 2015, Stincic* et al.* 2018), which are upstream modulators of mTOR (Martinez de Morentin* et al.* 2014*b*, Saxton & Sabatini 2017), makes them potential candidates to mediate the effects of E2 signaling on mTOR and, subsequently, its anorectic action.

In summary, our study shows that mTOR signaling in the ARC conveys E2’s anorectic effect. Our data also describe that hypothalamic mTOR pathway is of importance for understanding and potential treatment of the positive energy balance that characterizes states of estrogen deficiency, such as OVX or menopause.

## Supplementary Material

Supporting Figure 1

Supporting Figure 2

## Declaration of interest

The authors declare that there is no conflict of interest that could be perceived as prejudicing the impartiality of the research reported.

## Funding

This work did not receive any specific grant from any funding agency in the public, commercial, or not-for-profit sector.

## Authors’ contribution statement

I G-G, P B M M, A E-S and C C performed the *in vivo* experiments (OVX, peripheral, central and stereotaxic microinjections), the analytical methods (hormone measurements and Western blotting) and collected the data. A R-P validated the S6K1 adenoviruses. I G-G, P B M M, J F, R N, C D, M T-S, S T and M L designed the experiments, analyzed, discussed and interpreted the data. I G-G and M L made the figures. All authors reviewed and edited the manuscript and had final approval of the submitted version. M L developed the hypothesis, wrote the manuscript, coordinated and directed the project and secured funding.
