# Supplementary material for: mTOR signaling in the arcuate nucleus of the hypothalamus mediates the anorectic action of estradiol
Source: J Endocrinol. 2018 Jun 18;238(3):177–86. doi: 10.1530/JOE-18-0190 (PMC6055430; doi:10.1530/JOE-18-0190)
Supplement: Supporting Figure 1 [file joe-238-177-s001.pdf]

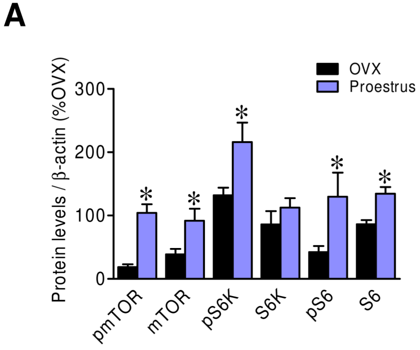

SUPPLEMENTAL FIGURE 1. Effect of proestrus on mTOR pathway within the ARC
(A) ARC protein levels of mTOR pathway of rats in proestrus or OVX rats. n=7 animals per group. All data are expressed as mean ± SEM. *P<0.05 *vs.* OVX.
