# Supplementary material for: mTOR signaling in the arcuate nucleus of the hypothalamus mediates the anorectic action of estradiol
Source: J Endocrinol. 2018 Jun 18;238(3):177–86. doi: 10.1530/JOE-18-0190 (PMC6055430; doi:10.1530/JOE-18-0190)
Supplement: Supporting Figure 2 [file joe-238-177-s002.pdf]

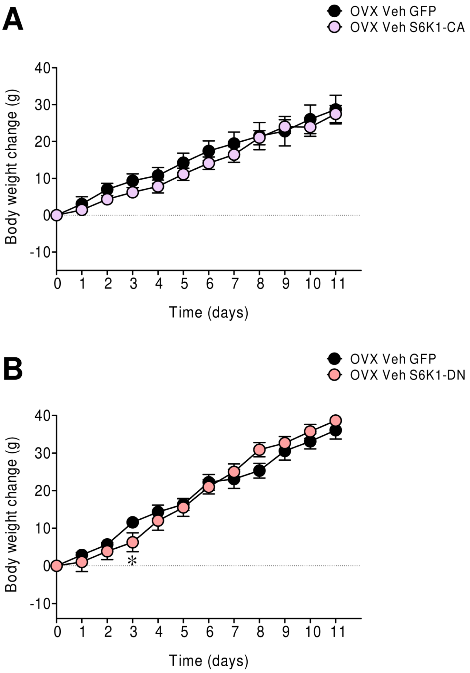

SUPPLEMENTAL FIGURE 2. Effect of activation and inhibition of mTOR pathway on control (vehicle-treated) OVX rats
Body weight change of OVX rats stereotaxically treated in the ARC with adenoviruses encoding **(A)** GFP or S6K1-CA or **(B)** GFP or S6K1-DN and SC treated with vehicle. n=8-9 animals per group. All data are expressed as mean ± SEM.
